# A large, paired dataset of robotic and handheld lumbar spine ultrasound with ground-truth CT benchmarking

**DOI:** 10.1038/s41597-025-06047-9

**Published:** 2025-11-10

**Authors:** Nicola A. Cavalcanti, Ruixuan Li, Laura Arango, Ayoob Davoodi, Kaat Van Assche, Yunke Ao, Aidana Massalimova, Mehrdad Salehi, Lukas Zingg, Tobias Götschi, Gianni Borghesan, Christoph J. Laux, Reto Sutter, Mazda Farshad, Matthias Tummers, Philipp Fürnstahl, Emmanuel Vander Poorten, Fabio Carrillo

**Affiliations:** 1 Research in Orthopedic Computer Science, Balgrist University Hospital, University of Zurich, Zurich, 8008 Switzerland; 2 Robot-Assisted Surgery Group, Department of Mechanical Engineering, KU Leuven, Leuven, 3001 Belgium; 3 University Spine Center Zurich, Balgrist University Hospital, University of Zurich, Zurich, 8008 Switzerland; 4 Spine Biomechanics, Balgrist University Hospital, University of Zurich, Zurich, 8008 Switzerland; 5 Core Lab ROB, Flanders Make, Leuven, 3001 Belgium; 6 Department of Radiology, Balgrist University Hospital, University of Zurich, Zurich, 8008 Switzerland; 7 Univ. Grenoble Alpes, CNRS, Grenoble INP, G-SCOP, 38000 Grenoble, France; 8 OR-X Translational center for surgery, Balgrist University Hospital, University of Zurich, Zurich, 8008 Switzerland

**Keywords:** Translational research, Preclinical research

## Abstract

Musculoskeletal disorders present significant socio-economic challenges globally, requiring innovative diagnostic and therapeutic approaches. Current intraoperative imaging techniques, including computed tomography (CT) and radiography, involve high radiation exposure and limited soft tissue visualization. Ultrasound (US) offers a non-invasive, real-time alternative but is underutilized intraoperatively, as it is highly observer-dependent. US enhanced by artificial intelligence shows high potential for observer-independent surgical guidance, decision support, and robot-assisted applications in orthopedics. Given the limited availability of high-quality, *in vivo* ultrasound data, we introduce a comprehensive dataset from a collection of handheld 2D US (HUS) and robot-assisted US (RUS) lumbar spine imaging in 63 healthy volunteers. This dataset includes demographic data, HUS and RUS imaging with synchronized 3D positioning data of the US probe, corresponding CT data, and CT-reconstructed 3D models of the lumbar vertebrae L1 to L5, establishing a robust baseline for machine learning algorithms. This extensive collection is the first anatomy dataset for the lumbar spine that includes paired CT, HUS, and RUS imaging, supporting advancements in intraoperative imaging and robot-assisted surgery.

## Background & Summary

An estimated 1.71 billion people worldwide are affected by musculoskeletal disorders, and there is a critical need for innovative diagnostic and therapeutic approaches^1,2^. Low back pain, fractures, and osteoarthritis are the most prevalent of these conditions, which together represent a significant health and economic burden, particularly in the context of an aging population^3–7^. This demographic trend increases not only the risk of disability but also the complexity of the orthopedic surgical interventions required^8–11^. The complex nature of human anatomy, combined with the critical proximity of nerves and blood vessels to surgical sites and the poor visibility of the region of interest, demonstrates the need for precise intraoperative methods to guide these procedures^12–14^.

Surgical navigation systems that rely on intraoperative computed tomography (CT) or the fusion of preoperative CT with intraoperative fluoroscopy remain the gold standard for intraoperative guidance^15–17^. While promising developments in “CT-like” MRI, based on spoiled gradient, zero-, or ultrashort-echo-time sequences with bone-signal post-processing, are emerging as radiation-free alternatives with comparable accuracy, these technologies are not yet widely adopted^18–21^. As a result, most current intraoperative navigation systems still depend on ionizing radiation, exposing both patients and healthcare professionals to potentially harmful levels that raise concerns about long-term health risks^22–24^.

Additionally, they are limited in their ability to adapt quickly to changes that occur during surgery^25–29^, and their poor visualization of soft tissues can hinder the identification and avoidance of critical structures, potentially leading to perioperative complications^30,31^.

Ultrasound (US) imaging is a promising alternative in this setting. Known for its non-harmful, real-time, and dynamic imaging capabilities, US has been extensively used for diagnostic purposes perioperatively^32–34^. Despite its potential, applying handheld 2D US (HUS) in surgical guidance remains limited to pre-clinical and early clinical research in orthopedics^35–39^. This limitation is due to several challenges: US is still predominantly regarded as a diagnostic rather than a therapeutic tool; significant advancements in automatic image segmentation are required to enhance its practicality; and the observer-dependent nature of current ultrasound systems discourages many professionals from adopting it for intraoperative use. Recent advances in robot-assisted 3D reconstruction of 2D US images represent a breakthrough in this research area^40–45^. Some US transducers are capable of real-time 3D imaging, but they currently suffer from lower imaging quality, structure insensitivity, and higher cost than 2D US transducers^46–50^. Therefore, the community mainly utilizes tracking- or robot-based odometry to create 3D US data by stacking 2D US images along the acquisition axis^51–53^. This development promises to overcome the limitations of traditional 2D HUS by providing comprehensive 3D visualization of anatomical structures, facilitating observer-independent use, and allowing orientation-independent imaging. Moreover, US-based 3D reconstruction could advance the perception of surgical navigation and robotic systems without the drawbacks of additional ionizing radiation.

This clinical trial aimed to produce a dataset for advancing machine learning applications of 3D reconstruction algorithms using HUS and robot-assisted US (RUS) data, critical for facilitating its translation from pre-clinical research to clinical application for surgical guidance^54^. One example of a potential downstream application in robot-assisted surgery is the FAROS project (EU Horizon 2020), aiming to leverage non-visual sensing technology to improve the autonomy of robotic pedicle screw drilling^55^.

Up to now, only a few musculoskeletal HUS datasets are openly accessible to the scientific community^56^, such as the Leg-3D-US datasets (44 volunteers)^57^, and a recent initiative, the TUS-REC challenge (100 volunteers)^58^, which aims to open-source a large HUS dataset of bilateral forearms with accurate positional information. To our knowledge, this is the first-ever RUS dataset of this size and topic made publicly available for large-scale scientific and clinical analyses^45,54^.

**Fig. 1:**
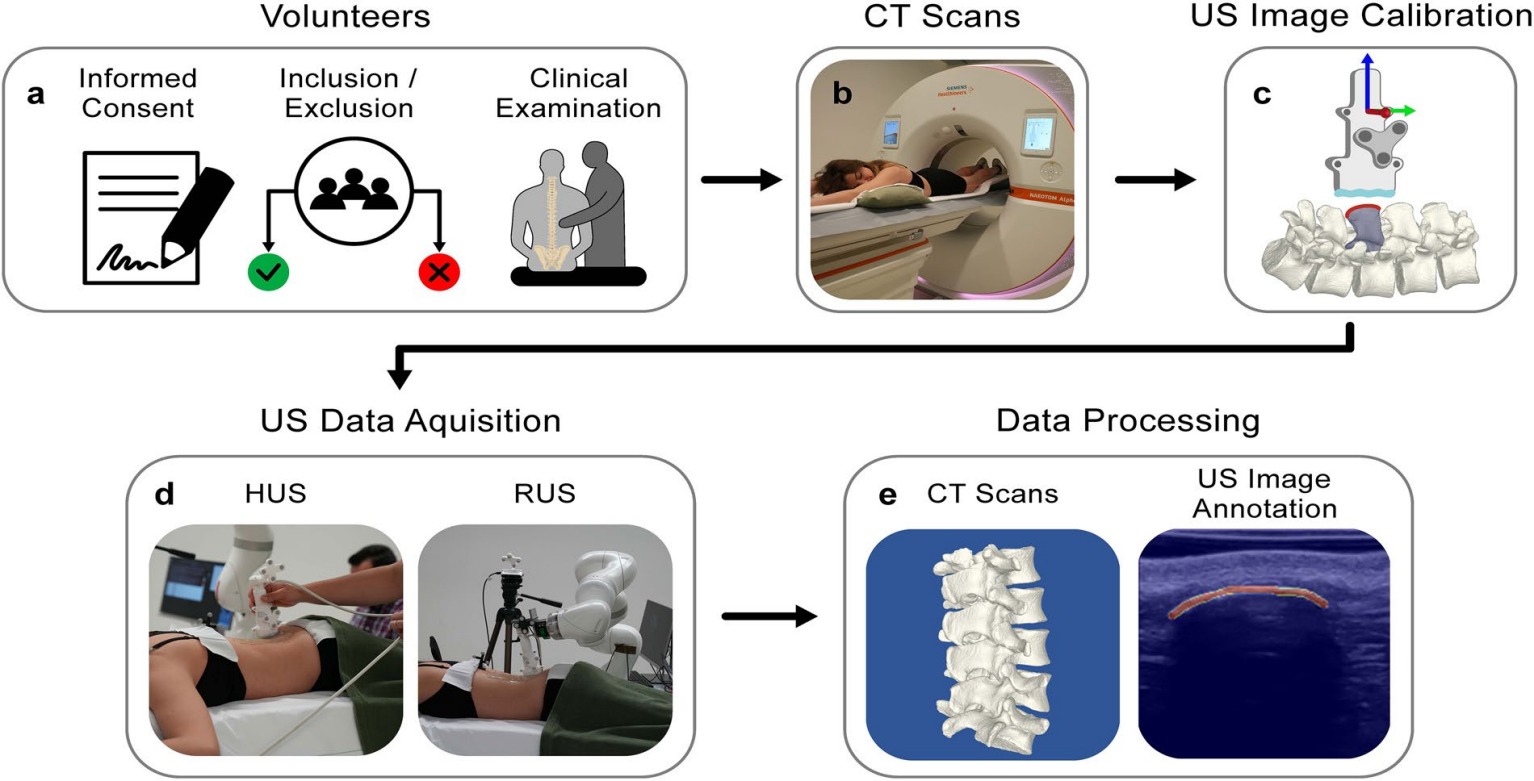
Study pipeline. (**a**) Sixty-three volunteers were recruited based on in-/exclusion criteria and passed a clinical examination of the lumbar spine. (**b**) An ultra-low-dose computed tomography (CT) scan was conducted. (**c**) The US setup was calibrated for accurate image recording. (**d**) Handheld (HUS) and robot-assisted US (RUS) scans were conducted. (**e**) Collected data was processed with the CT scan segmentation into 3D surface models and manual annotation of the bone surface in 2353 HUS and 3738 RUS images.

We present a comprehensive dataset from a single academic spine center, including demographics, HUS, RUS, and high-quality, ultra-low-dose CT images of the lumbar spine of 63 healthy volunteers^54^. Figure 1 provides an overview of the study pipeline, from volunteer selection and eligibility criteria to the different imaging methods and data processing. As such, this dataset adds to the existing collection of lumbar spine medical imaging data and provides a foundation for developing advanced intraoperative strategies in managing musculoskeletal disorders using RUS for surgical guidance^54,59–61^. Moreover, this ultrasound dataset complements the recent works published on medical imaging data descriptors for CT, MRI, and Finite Element Model data of the lumbar spine, adding more opportunities for machine learning approaches^54,62–64^.

## Methods

The dataset of this study was obtained through the study pipeline described in Fig. 1, which encompasses the following steps:

- First, all participants’ demographic information, including sex, age, height, weight, and BMI, was collected (see section Volunteers).
- Afterward, a CT scan of the lumbar spine was performed on the same day, right before the US scans (see section Computed Tomography Scans).
- Following, paired ultrasound scans were acquired using two separate setups on each participant (see section Ultrasound Data Acquisition).
  - The system was previously calibrated for accurate image recording (see subsection Ultrasound Image Recording and Processing).
  - 223 HUS scans were performed (see section Handheld Ultrasound Scans).
  - 375 RUS scans were performed (see section Robot-assisted Ultrasound Scans).
- Finally, the obtained data were processed for accurate storage and further use (see subsection Data Processing).
  - All CT scans were segmented into 3D surface models (see section Computed Tomography Image Processing).
  - The bone surface was annotated in 2353 HUS and 3738 RUS frames (see section Data Annotation and Labeling).

### Volunteers

For this study, a total of 63 healthy adults (39 females, 24 males) aged 20–35 years (mean 25 years) with a BMI of 19–26 kg/m^2^ (mean 22 kg/m^2^) were recruited. Healthy adults were chosen to ensure motion-free scanning protocols, satisfy the ethical constraints of ultra-low-dose CT, and create a publicly shareable reference dataset of healthy paired US and CT images^54^. A detailed overview of the demographic characteristics is available in the repository. Oral and written informed consent were obtained from all volunteers before data collection for the study. Additionally, all participants consented to further use of their data for research and photography publication when applicable. Limiting the cohort to healthy volunteers also enabled the completion of all acquisitions within a one-month window, which was budgeted for the trial data collection, allowing for the scheduling of participants back-to-back. This approach would have been impractical for patient recruitment. Future work will involve pathological cohorts (e.g., vertebral fractures, osteoarthritis), where altered anatomy and potential discomfort may require customized positioning and imaging settings. The institutional review board and the local ethics committee of the Canton of Zurich, Switzerland, approved the study protocol (BASEC No: 2023-00350, 18.04.2023). Inclusion and exclusion criteria assessed the eligibility of the volunteers (Table 1). A study-specific clinical examination of the lumbar spine was performed on all volunteers to confirm eligibility, and pregnancy tests were conducted when applicable. All imaging data were collected between May and June 2023 at the University Hospital Balgrist, Zurich, Switzerland. Demographic data and inclusion/exclusion criteria were collected and managed using REDCap electronic data capture tools hosted at Balgrist University Hospital^65,66^. This study was registered as a clinical trial on ClinicalTrials.gov (Trial No: NCT05904418, 03.05.2023).

**Table 1:** Inclusion and exclusion criteria for participation eligibility.

Inclusion	Exclusion
Oral and written informed consent from the volunteer	Documented volunteer refusal
Volunteers aged ≥18 years and ≤35 years	Volunteers for whom CT cannot be performed
BMI ≥19 kg/m^2^ or ≤30 kg/m^2^	Positive pregnancy test before radiology (contraindication to CT)
Proficient in German or English	Pregnancy
	Chronic pain in the lumbar spine
	Moderate or severe deformity of the lumbar spine
	Any prior intervention on the lumbar spine • Chiropractic adjustment therapy • Injections such as local anesthetics and corticosteroids • Surgery
	Fracture of the lumbar spine

### Data acquisition logistics

Given the collaborative nature of the project between KU Leuven and the University of Zurich, a substantial and coordinated effort was essential to ensure the quality and comprehensiveness of the dataset. Six months of protocol design, ethics approval, and pilot testing preceded a focused one-month on-site campaign at the University of Zurich, during which all 63 volunteers were scanned (≈ 2 h per participant; up to four participants per day). Six researchers staffed each on-site session: two medical personnel who managed recruitment performed the clinical examination and oversaw the ultra-low-dose CT acquisition with the assistance of a CT technologist, and a team of four to five members was responsible for protocol adherence, HUS imaging, real-time data logging, RUS scanning, and volunteer safety monitoring. Following the acquisition, over 15 months were spent processing, validating, and curating the CT and US datasets^54^.

### Computed tomography scans

**Fig. 2:**
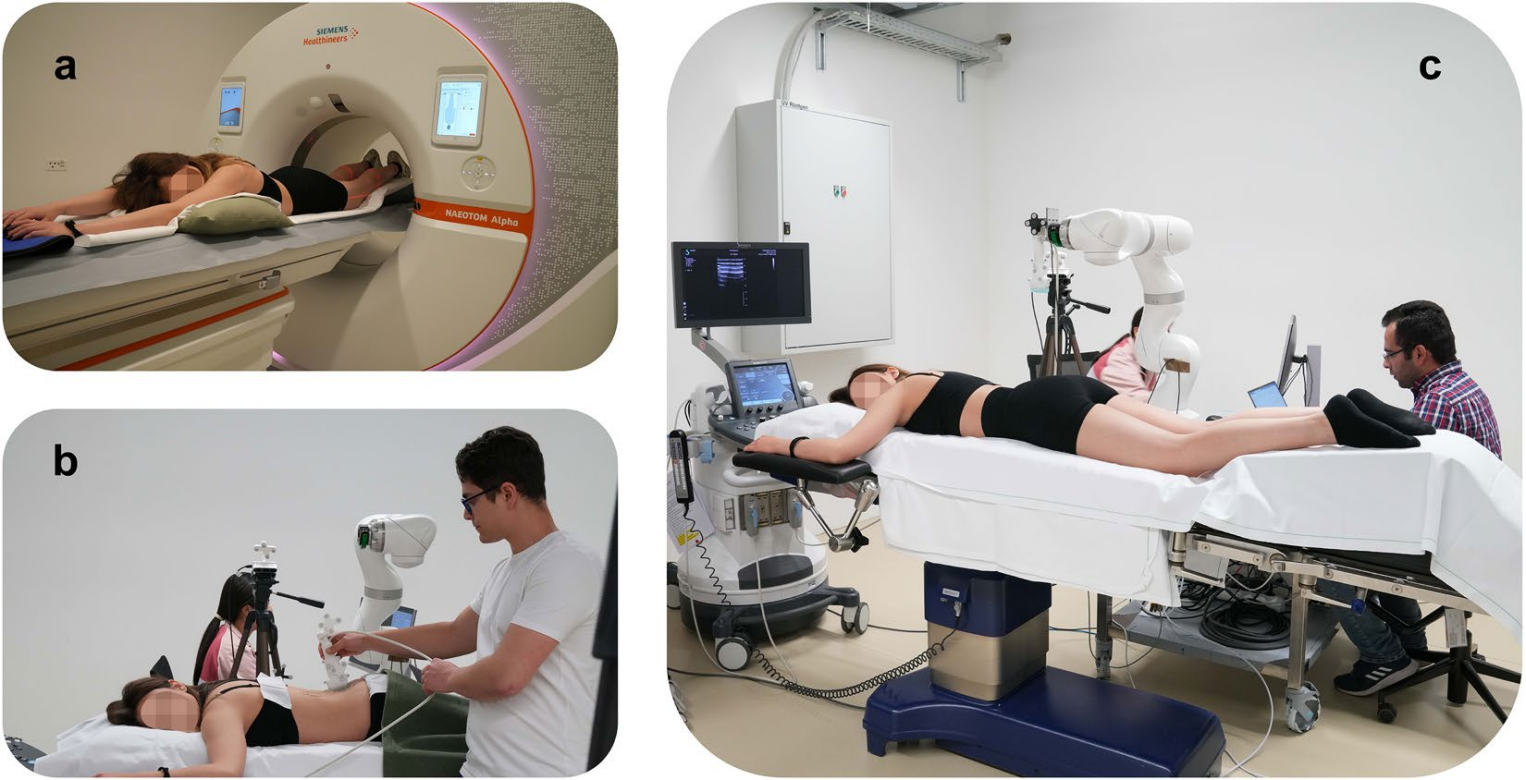
Experimental CT, HUS, and RUS scan setup. The volunteers were positioned prone on the table during (**a**) CT Scan, (**b**) HUS, and (**c**) RUS. In all three imaging examinations, the arms were rested above the head, and a positioning cushion supported the feet and lower legs for comfort. The US device was positioned at the head of the volunteer. The HUS and RUS scans were conducted in the same tracking space, with the camera placed approximately two meters away on the left side of the volunteer.

Each participant underwent a tin-filtered ultra-low-dose (ULD) CT scan (NAEOTOM Alpha, Siemens, Erlangen, Germany) of the lumbar spine, including the vertebrae L1 to L5 and partial views of the iliac bones^67^. The CT data were obtained using a specific acquisition protocol developed for this study. During this imaging procedure, the volunteers were positioned prone with their heads on a pillow and their arms above their heads (e.g., Fig. 2a). Further, a structured positioning cushion (MRI Knee support cushion, Siemens, Erlangen, Germany) was placed below the lower legs and ankles to have a similar comfortable position of the spine during the CT scan as in the subsequent US examinations (e.g., Fig. 2b,c). Afterward, a CT topogram and axial reconstructions of 0.6 mm and 2.0 mm slice thickness and increments with a matrix size 512 × 512 were collected, which are the current standard for ULD scans at our institution (Table 2)^67,68^.

**Table 2:** Custom CT acquisition protocol.

CT Acquisition Protocol	
Device model name	NAEOTOM Alpha
Scan mode	QuantumSn
Tube potential (kVp)	140
Tube current (mAs)	134 (Manual)
Collimation (mm)	144 × 0.4
Pitch factor	0.8
Rotation time (seconds)	0.5
Iterative reconstruction algorithm	QIR
Algorithm strength	3
Reconstruction kernel	Br60
Slice thickness (mm)	0.6/2.0
Slice increment (mm)	0.6/2.0
Matrix size	512 × 512
Field of view	120 × 160
Mean CDTIvol (range; mGy)	3.29 (2.62–4.27)
X-Ray filter material	Tin

### Ultrasound data acquisition

**Fig. 3:**
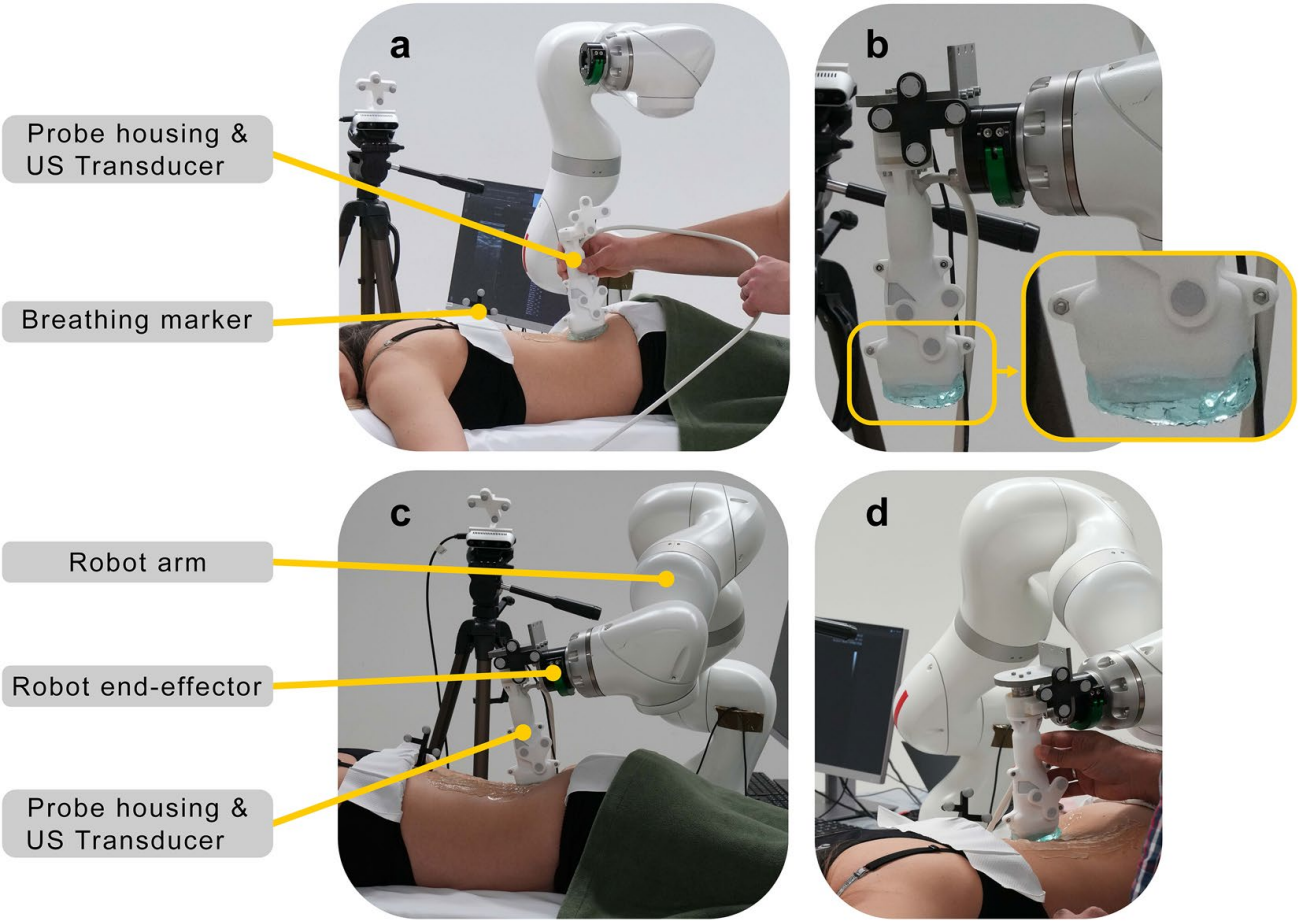
In detail, the two separate experimental setups of the HUS and RUS scans. The transducers were housed in a custom-made cast featuring two markers for optical tracking. (**a**) The investigator on the volunteer’s left side conducted the HUS scans. (**b**) The custom-made robot end-effector included a 6-DOF force-torque sensor, and for both RUS and HUS scans, a gel pad was attached to the transducer’s tip (picture-in-picture). (**c**) The robotic system was positioned on the volunteer’s right side. (**d**) The physician manipulates the robotic arm to define the scanning area on the patient’s lower back using admittance control.

Two different US acquisition setups were performed: HUS and RUS. 223 HUS and 375 RUS scans could be completed in the data collection without errors or data-saving issues. The total number of HUS and RUS scans differs mainly because the “Perpendicular”-type US scans were repeated three times for each RUS scan. This approach was chosen because the perpendicular scans are the simplest for the system to execute, making them less prone to errors. Additionally, this type of scan represents the current standard for our scanning system to achieve sufficient bone surface reconstruction, ensuring reliable and consistent data collection^52^. The US section of the repository provides an overview of the availability of HUS and RUS scan types. All US imaging was acquired using a clinical US device (Aixplorer Ultimate, SuperSonic Imagine, Aix-en-Provence, France; Fig. 2c). The volunteers were positioned prone with arms folded above the head on a height- and tilt-adjustable operating table (Maquet Alphastar, Getinge AB, Göteborg, Sweden). A structured support cushion was placed beneath the lower legs and ankle to comfort the volunteers during the scans and to create the same body position as during the preceding CT scan (e.g., Fig. 2c). The participants were instructed to remain stationary, breathe slowly, and relax. The US data were collected using a linear transducer (SuperLinear™ SL10-2) set to 10 MHz. The image depth (7.7 cm) and focus (4 to 7 cm, slightly adjusted for each volunteer) were previously assessed and defined by an expert radiologist for this custom imaging protocol. The gain or brightness of the US image was adjusted between 60% and 74%, depending on the volunteer and soft tissue thickness. Two identical transducers were available for the HUS and RUS scans, respectively (e.g., Fig. 2b,c). The transducers were embedded in a custom-made 3D-printed transducer housing (material PA-12, Formiga P110, EOS GmbH, Munich, Germany) featuring markers with individual geometries of reflective disks (Atracsys LLC, Puidoux, Switzerland) for optical tracking (e.g., Fig. 3a,b). An additional marker was positioned on the midline of the volunteer’s spine to monitor chest movements during breathing (e.g., Fig. 3a,c). The US images were recorded as 1080 × 1920 pixels by a frame grabber (Epiphan, Palo Alto, U.S.A.) at 15 Hz.

**Fig. 4:**
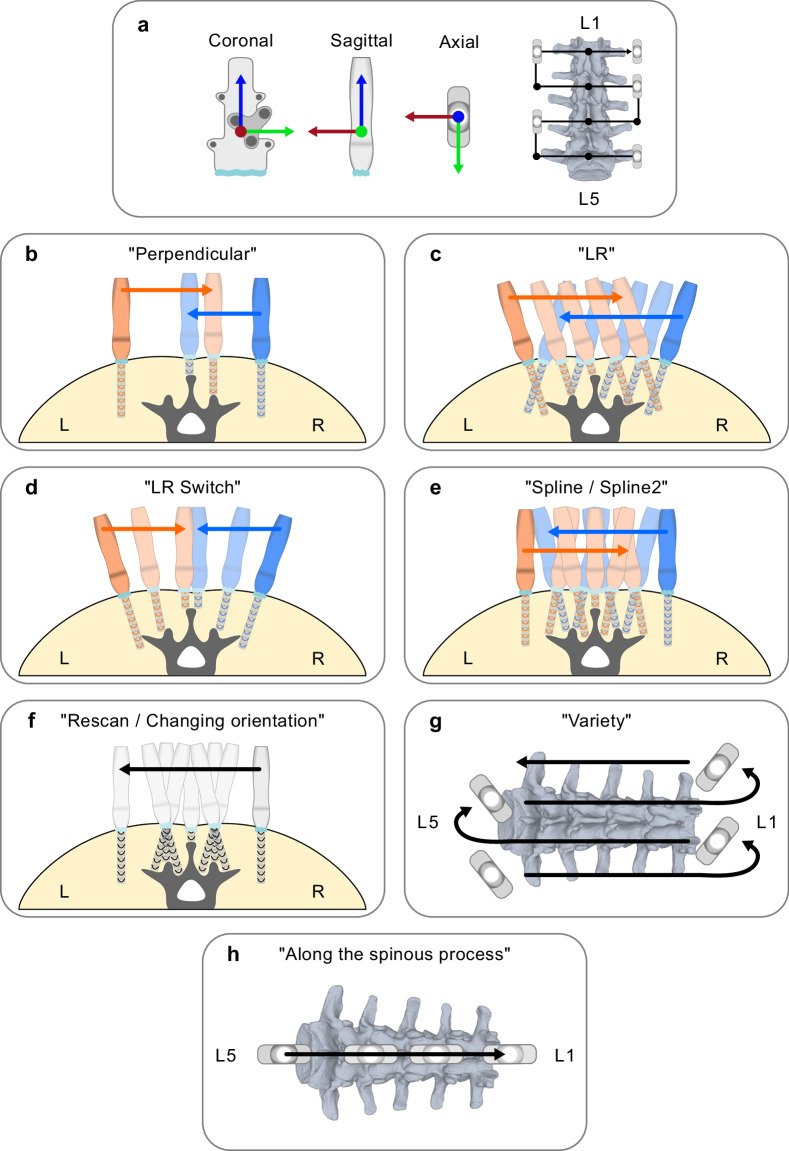
Multiple types of HUS (**b** to **c**, **f** to **g**) and RUS (**b** to **f**, **h**) scans were completed. (**a**) The coordinate system of the US transducer in its coronal, sagittal, and axial views is shown with the x-, y-, and z-axis illustrated in red, green, and blue, respectively. All US scans except (**g,h**) start at the right side of the vertebra L5, shown with the transducer parallel to the lumbar spine in grey from the top view. The transducer then travels along the 12 predefined points (black) on the scanning path (black) cranially toward the vertebra L1. (**b–e**) A torso and the lumbar vertebra L3 are illustrated with the transducer movement pattern from an axial view. Forward and reverse scanning paths are illustrated in orange and blue, respectively. (**f**) Anatomy is illustrated as in (**b–e**) The US transducer travels forward from right to left, tilting +20° to −20° above critical structures to follow the anatomical shape of the vertebra. (**g**) As the starting point, the transducer is positioned at a diagonal 45-degree angle to the lumbar spine on the right side of vertebra L5 (grey). When reaching the most cranial and caudal scanning positions at vertebrae L1 and L5, the transducer is turned 90° toward the spine. (**h**) The transducer travels from below the spinous process of vertebra L5 in a straight motion just above the spinous process of vertebra L1.

The coordinate system of the US transducer is illustrated in Fig. 4a, with the US transducer advanced along its x- (red) and y-axis (green). During RUS scanning, a constant force of 5 N was maintained along the probe’s z-axis (blue) to ensure optimal image quality with limited tissue displacement. The force was measured only during RUS scanning, as it was not feasible to measure it during the HUS scans without meaningful hardware extension and limitation of the traditional HUS scan process. However, the physician conducting the HUS scans was carefully instructed to perform the HUS scans with a similar force in the z-axis. This force was achieved through a hybrid control strategy, aligning the z-axis with the surface normal vector computed from a predefined trajectory derived from the physician’s manual point selection on the volunteer’s lower back^52^. Real-time control was facilitated by OROCOS (Open Robot Control Middleware) and eTaSL (expressiongraph-based Task Specification Language)^69,70^. The robotic US system has different safety levels implemented at the software and hardware levels. The safety hierarchy goes from software-based low-level to the hardware-based safety button.

#### Ultrasound image recording and processing

For accurate image recording, temporal and spatial calibration were performed using a custom-designed Z-phantom with the proposed robotic system before image acquisition^71^. This calibration yields the transformation between US images and the robot end-effector, enabling their synchronization with the end-effector poses. The transformation and calibration are explained in detail below (see section technical validation; spatial tracking and Fig. 7a). The pairing of the US probes in both HUS and RUS was achieved using optical tracking with a Fusiontrack500 camera (Atracsys LLC, Puidoux, Switzerland), positioned approximately two meters away on the left side of the volunteer. The positioning of the camera was marked on the ground for reproducibility during the entire data collection, and before each data collection day, the camera system was calibrated. A gel pad (Focus pad, aiSon™ Technologies, Benglen, Switzerland) was attached to the US transducer to ensure consistent skin contact without extensive pressure on the tissue (e.g., Fig. 3b; picture-in-picture).

#### Handheld ultrasound (HUS) scans

A physician first palpated the pelvis and the spinous processes of the thoracic and lumbar vertebrae T12 to L5 to mark the anatomical area of interest for the subsequent US scanning. Then, the same physician confirmed the marked area with a preliminary HUS scan of the spinous and transverse processes. After that, at least three standardized and one optional scan HUS in an “S”-shaped scanning pattern were conducted on the lumbar spine for the data collection. The standardized scans included the “*Perpendicular*,” “*Rescan*,” and “*LR*”, while the optional scan was labeled “*Variety*” (e.g., Fig. 4b,c,f,g). A total of 71, 62, 61, and 29 scans, respectively, were completed for these categories. All standardized scans started on the right dorsal side of the pelvis, laterally to the right transverse process of the L5 vertebra, and progressed medially across the spine to the left side of the respective vertebrae. The “S”-shaped scan patterns advanced cranially toward the vertebra L1 in 3 to 4 cm increments.

The first US scan (e.g., Fig. 4b), “*Perpendicular*”, was completed with the US transducer perpendicular to the skin. For the second scan, “*Rescan*” (e.g., Fig. 4f), the investigator took the anatomical shape of the vertebrae into account and scanned back-and-forth in approximately 20-degree angles to the left and right on critical structures, such as the spinous, mammillary, and transverse processes. The focus on the anatomical shape puts this scan type closer to the clinical use case. Previous work has shown that the maximal angle of tilting is 35 degrees, and the most commonly used angle is 10 degrees tilt to either side^72^. Preliminary tests of our consortium have shown that 20-degree angles allowed us to scan the variable spinous bone surface with optimal imaging results and skin-to-probe contact^73–75^. The third scan (e.g., Fig. 4c), “*LR*” or left to right, was started by tilting the US transducer at a 20-degree angle to the right. When the other side’s transverse process was reached, the US transducer was tilted to the left at the same angle, and the scanning was repeated in reverse order. Then, the same increments were used to move toward the upper spine as in the previous scans. A fourth scanning path (e.g., Fig. 4g), “*Variety*”, was conducted only in a subset of the volunteers due to time limitations. The path started in the same location as the three standardized HUS scans used to scan the lumbar spine up and down. However, the US transducer was positioned at a diagonal 45-degree angle to facilitate the optical tracking from the same camera position. Therefore, the US transducer had to be turned 90° toward the spine when reaching the most cranial and caudal scanning positions. A custom program with a graphical user interface (GUI) was used to store the collected data. The HUS scans took 3 minutes on average, generating 2700 images per scan.

#### Robot-assisted ultrasound scan

After the HUS scans were completed, the volunteers remained in the same anatomical position for the subsequent RUS scans. All RUS imaging was acquired using a research system previously described and developed within a European collaboration project (FAROS)^52,55^. The proposed framework integrates automatic robotic scanning with US image and probe position recording.

The housing of the transducer for the RUS system included a 3D-printed segment incorporating a 6-DOF force-torque sensor (Nano25, ATI Industrial Automation, Apex, U.S.A.), to which another marker was affixed (e.g., Fig. 3b) to ensure the capture of any movement between the US probe and robot arm.

Before scanning, the physician manually selected twelve predefined points on the patient’s lower back using admittance control (e.g., Fig. 4a). These points define an “S”-shaped trajectory for the robotic arm during automated scanning. With the anatomical area of interest marked, it was checked whether the area was within the workspace limits of the robotic arm holding the probe. If necessary, the position of the surgical table was adjusted (e.g., Fig. 2a).

For each subject, three consecutive “*Perpendicular*” scans were conducted for the RUS scan, as done previously with the HUS (e.g., Fig. 4b). Then “*LR*”, “*LR switch*”, “*Spline*”, “*Spline2*”, “*Changing orientation*”, and “*Along the spinous process*”-type scans were performed, which due to time constraints were performed in 45, 54, 24, 27, 25, and 11 cases, respectively (e.g., Fig. 4c–f,h). These scanning paths were automatically generated based on three waypoints along each vertebra: (1) at the start of the transverse process, (2) at the center of the spinous process, and (3) at the end of the other transverse process. Most RUS scans started in the same position as the HUS scans, on the right dorsal side of the pelvis, lateral to the right transverse process of the L5 vertebra (e.g., Fig. 4a).

In the “*LR*” path, the probe scans the full vertebra from the right to the left transverse process at a constant 20 degrees and returns in the same path at a constant −20 degrees (e.g., Fig. 4c). The decision on 20-degree angles is the same as mentioned above for the HUS scans. In the “*LR switch*” scan, the US probe starts at the right transverse process. It remains at a constant angle of 20 degrees until the center point at the spinous process (e.g., Fig. 4d). At the center, it rotates to −20 degrees and scans at this constant angle until the left transverse process has been entirely scanned. The “*Spline*” scan simulates a scan path following the shape of the vertebrae (e.g., Fig. 4e). The scan followed an estimated shape to position the US probe as perpendicular as possible to the bone surface with a variable inclination of a maximum of +20 or −20 degrees as the scan progresses. The “*Spline2*” scan was introduced as a modified version of “*Spline*” (e.g., Fig. 4e), in which the inclination of the US probe gradually increases from 0 degrees at the right transverse process up to 20 degrees at the spinous process. This motion is then mirrored: the US probe inclination is reduced to −20 degrees at the spinous process and then gradually increased to 0 degrees inclination at the left transverse process.

The “*Changing orientation*” path followed a scanning trajectory such that the US probe was almost perpendicular to the underlying vertebral bone surfaces, similar to the HUS “Rescan” scan type (e.g., Fig. 4f). The trajectory for this path was determined by an algorithm based on information of the vertebra bone surface outline obtained from previously the “*Perpendicular*” scan of the same volunteer^73^. In the path “*Along the spinous process*”, the US probe is initially positioned on the spinous process of L5 in a transverse spinous process view and moved along the longitudinal axis of the spine, scanning over all the spinous processes of the lumbar vertebrae (e.g., Fig. 4h). The RUS scans took 4 to 5 minutes on average, generating 3600 to 4500 images per scan.

### Data processing

#### Computer tomography image processing

As previously described, the vertebrae (L1-S1) in the CT Scans were manually segmented with global thresholding and the region-growing tool in a medically certified segmentation software (Materialise Mimics, Leuven, Belgium)^76–79^. The 3D surface models are then exported as STL files. Each image segmentation was subject to review and refinement by a physician.

#### Data annotation and labeling

The physician (N.A.C.) responsible for collecting the HUS images performed the US data annotation process to reduce the potential for information loss between the US scanning and annotation phases. Annotation was performed on the raw images from the previously mentioned frame grabber. The (.raw) image format was purposefully chosen since it is timestamped. The primary objective of the annotation process was to identify all visible bone surface pixels and ensure the creation of a diverse and representative annotated dataset^54^. To achieve this, we randomly selected nine subjects for annotation, with seven subjects having both HUS and RUS scans annotated, while two had only RUS scans annotated. For the HUS scan types, annotations were performed on a subset of frames, which included one “*Perpendicular*”, three “*LR*”, two “*Rescan*”, and one “*Variety*” scan type, chosen randomly to capture variability in imaging conditions. Similarly, for the RUS scan types, annotations were performed on seven “*Perpendicular*”, three “*LR switch*”, and one “*Spline2*” scan type, also randomly selected to ensure diversity in the annotated dataset^54^. 2353 HUS and 3738 RUS frames were annotated to obtain a diverse annotated dataset^54^. In each scan, a minimum of 300 frames of critical anatomical features, including the spinous, mammillary, and transverse processes, were manually annotated. Ultimately, 6091 annotated frames were generated, constituting a comprehensive dataset for training a 3D image segmentation network such as the U-Net used for this study’s US reconstructions^54,56,73^. The variability in the number and types of scans annotated was necessary to account for the diverse imaging scenarios and to optimize the dataset for robust model generalization^54^.

**Fig. 5:**
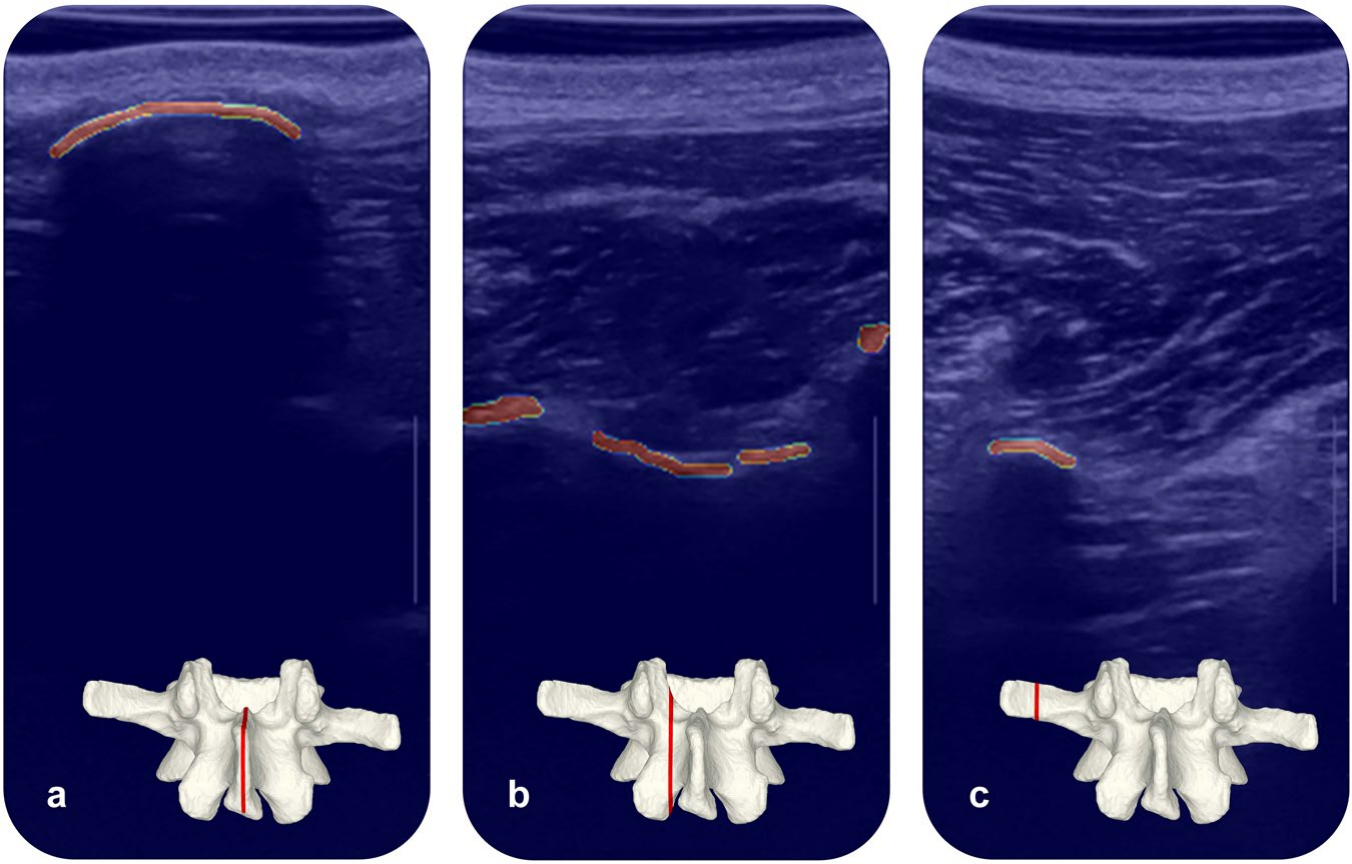
Labeled bone surfaces and their approximate location on an L3 lumbar vertebra 3D surface model. (**a**) The spinous process, (**b**) a lamina, and (**c**) a transverse process are illustrated in red in cropped 2D US images. Compared to the 3D surface model, the US images can show discontinuities in the labeled bone surface due to partial uncertainty in identifying the bone surface (Example **b**).

The annotations encompass each distinct bony anatomical feature observed in the visible vertebra (e.g., Fig. 5a–c). All frames are concomitant with the bone structure and, therefore, within the anatomical region of interest.

The annotation was conducted using Labels© (ImFusion GmbH, Munich, Germany). The initial step involved uploading all Portable Network Graphics (.png) images of a participant’s predefined US scan into the software database. Subsequently, the bone surfaces within the imaging frames were identified and labeled with the annotation “bone surface”. The ultrasound images and the corresponding labels are exported in raw (.raw) and MetaImage (.mhd) formats, respectively. Moreover, the mapping of the images to the image origin is available in a data list (.txt) file.

## Data Records

**Fig. 6:**
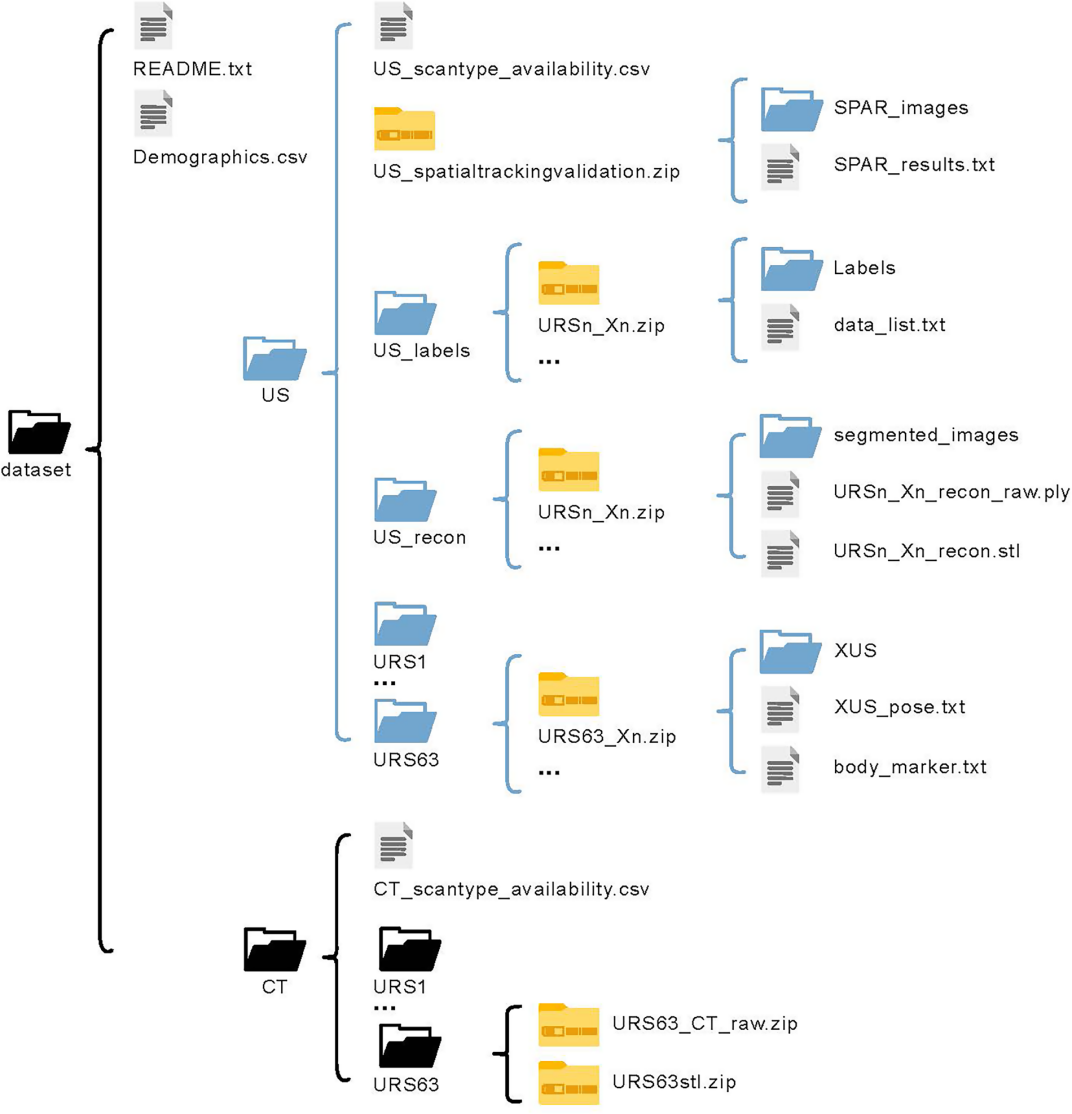
Overview of the repository’s data structure. The dataset^54^ contains a readme file, a demographics overview, and separate US (light blue) and CT (black) data. Within the US and CT folders, each volunteer folder can be found. All available Hn-US, Rn-US, and CT data in each volunteer folder are in zipped format (yellow). Each X(H/R/D/M)n-US zip file contains the US images and the pose information from optical tracking from the handheld US transducer, robot end-effector, or breathing marker, respectively. The CT folder includes information on CT scan type availability, and each CT volunteer folder contains a zip file of the raw CT images and the corresponding 3D surface models. The US folder includes several key components: the scan type availability, the zipped spatial tracking validation files along with the scan-path area ratio (SPAR) images, and a data list. It also contains the US recon folder, which holds the segmented US images and reconstruction point clouds (both with and without outliers). The US label folder also contains zip files for each labeled US scan, including the labels and a corresponding data list.

The complete dataset is publicly accessible at 10.48804/3XPCAE, with its structure illustrated in Fig. 6^54^. Users should select the “Tree” view rather than the “Table” view to display the folder structure correctly. A readme file provides metadata for the data repository, and a comma-separated values (.csv) file contains the demographic characteristics of the volunteers. The uncompressed folder includes the data from two imaging modalities: CT and US. The CT folder includes CT_scantype_availability.csv, which details image dimensions, resolution, and available protocols. The CT data are available in raw format as Digital Imaging and Communications in Medicine (DICOM or .dcm) files, accessible via a zip file. The 3D segmentation models are provided as Stereolithography (.stl) files. Data for each volunteer is stored separately under the naming convention URS*n*, where *n* represents the volunteer number. The US folder contains US_scantype_availability.csv (including image dimension, resolution, and available protocols), a zipped folder of the US spatial tracking validation, and the volunteer folders with the zipped US scan data. Additionally, the US folder includes a US labels subfolder with zipped files of labeled US images and a US recon subfolder with zipped files of US bone surface reconstructions.

Each zipped file of the US scan data contains the following information for the ultrasound scans: (1) a folder containing the unprocessed US images, (2) a (.txt) file with the tracked poses of the US images, and (3) a (.txt) file with the tracked poses of the body marker for breathing. Each volunteer’s unzipped folder contains all handheld and robotic scanning data stored in separate zip files. The handheld scans are designated as “H*n*.” In contrast, the robotic scans are abbreviated as “R*n*” for the “*Perpendicular*” and “D*n*” for all other scan types except the “*along the spinous process*”- type, which is labeled “M*n*”. In all these labels, *n* represents the trial number.

These folders contain all of the recorded US images of the respective scans. The data obtained from US imaging are available in (.png) format. Handheld scan images are stored in the “HUS” folder. In contrast, robotic scan images are distributed across the “RUS,” “MUS,” and “DUS” folders, corresponding to “*Perpendicular*”, “*Along the spinous process*”, and all other robotic scanning types, respectively. The images are arranged sequentially in temporal order.

The file “HUS_pose.txt” documents the poses of each US image captured via the optical tracking system, with the optical marker pose $T_{M}^{C}$ stored as x, y, z, qx, qy, qz, qw, and timestamp.

**Fig. 7:**
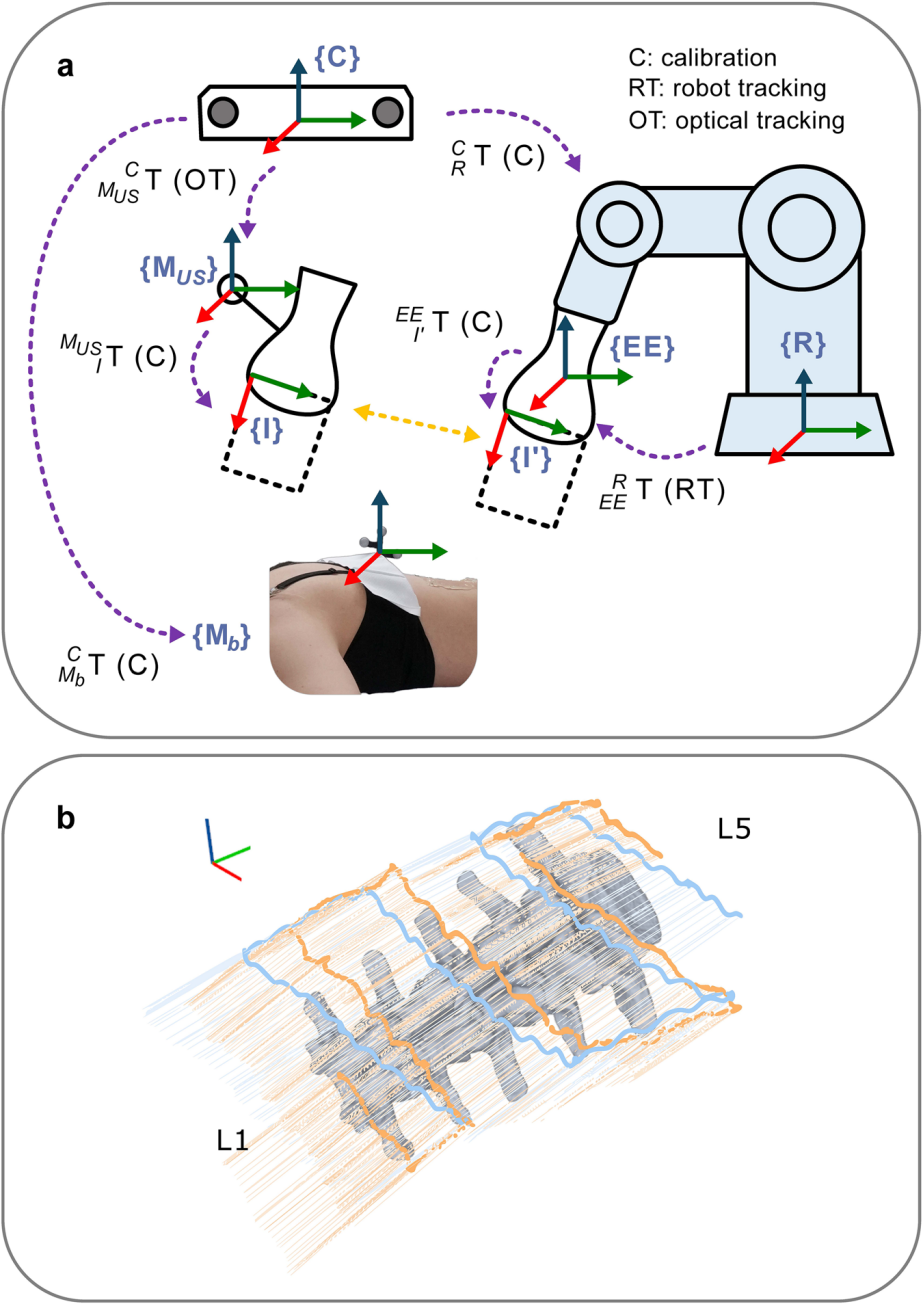
Spatial tracking method and signals of the HUS and RUS scans. (**a**) The transformations of **T** and the coordinate frames of the proposed systems. (**b**) Visualization of the scan trajectories of a HUS scan (orange) and a RUS scan (blue) acquired on the same volunteer. Thick lines represent the top-left corner of the ultrasound image frames, while fine lines indicate the top edge of the generated images, projected along the spine from L1 to L5. The coordinate system axes are shown with red (x), green (y), and blue (z). The scan-path area ratios (SPAR) quantify the cranial-caudal scan relative to the length of the L1-L5 CT model: 1.124 for the RUS scan and 1.239 for the HUS scan, indicating slightly greater scan coverage with the handheld scan in this example.

Similarly, the files “RUS_pose.txt”, “MUS_pose.txt”, and “DUS_pose.txt” record the robot end-effector poses $T_{{EE}}^{R}$, stored as x, y, z, roll, pitch, and yaw. These poses are synchronized with the US images during acquisition through timestamp comparison (e.g., Fig. 7a). The “body_marker.csv” file contains measurements of the optical marker attached to the volunteer’s back, with the pose $T_{{M}_{b}}^{C}$ recorded as x, y, z, qx, qy, qz, qw, and timestamp.

## Technical Validation

### Spatial tracking

The process of validating the spatial tracking data involves comparing the data obtained from the optical tracking camera (HUS scans) with that obtained from robotic tracking (RUS scans). Specifically, we compare the estimated poses of US image frames between HUS and RUS scans. The coordinate systems used are illustrated in Fig. 7a and include the following frames: optical camera {*C*}, HUS probe marker {*M*_*US*_}, HUS image {*I*}, breathing marker {*M*_*b*_}, robot base {*R*}, robot end effector {*EE*} and RUS image {*I*’}. The transformations $T_{R}^{C},T_{I{\prime} }^{{EE}}$ and $T_{I}^{{M}_{{US}}}$ are calibrated before the scans using a custom-designed phantom. From robot tracking data and optical tracking data, we could obtain $T_{{EE}}^{R}$ and $T_{{M}_{{US}}}^{C}$, respectively, for each US frame. Finally, we compared $T_{I}^{C}=T_{{M}_{{US}}}^{C}\cdot T_{I}^{{M}_{{US}}}$ and $T_{I{\prime} }^{C}=T_{R}^{C}\cdot T_{{EE}}^{R}\cdot T_{I{\prime} }^{{EE}}$.

An analysis of the tracking signals is provided in the repository, and their scan-path area ratio (SPAR) is assessed. For every scan conducted, the dataset includes measuring the scanned area swept by the tracked probe over the dorsal vertebral anatomy between L1 and L5, starting from the right dorsal side of the L5 vertebra^54^. SPAR is defined as the ratio between this swept area and the corresponding area in the y direction of the L1-to-L5 CT segmentation; a value of 0% indicates no anatomy was scanned, whereas 100% signifies a complete overlap. We calculated this area as the displacement along the y-axis between the most superior and most inferior positions in the tracked poses of the ultrasound image frame. Likewise, we determined the length of the L1-L5 CT model by measuring its extent along the y-axis. We then evaluated the ratios between the covered distance and the length of the L1-L5 CT model for both handheld and robotic scans. The resulting SPAR for handheld scans is consistently greater than or equal to 85%. The SPAR is more variable for robotic than handheld scans, ranging from 54% to 100%. Generally, it is critical to note that the SPAR of HUS and RUS are inherently different due to the scanning speed of the probe, which was intentionally restricted for the RUS due to the safety and comfort of the volunteers. Therefore, the slower speed resulted in lower SPAR values due to study time constraints.

Meanwhile, the standardized “*Perpendicular*” type RUS scans had SPAR values similar to those of the HUS scans. More complex and variable RUS scans, such as the “*Changing orientation*”-type scan, resulted in a lower SPAR but could result in improved segmentation possibilities. SPAR provides a registration-free indicator of scan completeness that can be employed to select scans suited to individual workflows. Additionally, the dataset contains figures visualizing the sweep trajectories for both scanning methods (e.g., Fig. 7b)^54^. These are named “URSn_Xn_SPAR.png”, with *n* representing the volunteer and trial number, and X specifies the scan type (H/R/D/M). They can be found in the “US_spatialtrackingvalidation” folder along with a “SPAR_results.txt” file noting the length ratio of the respective scan versus the length of the L1-L5 CT model.

### CT scans and segmentation

CT scans were generated using a clinical-grade CT and a clinical imaging protocol. An expert spine surgeon and a senior radiologist reviewed the raw and segmented CT data, and an orthopedic resident reviewed all imaging data. After a full review, all collected data were considered good quality for inclusion, and none were excluded.

### US scans and segmentation

Due to the size of the dataset, it was not possible to provide segmented data for all US images and scans^54^. However, as indicated in Fig. 6, the data repository in the US recon folder provides examples of reconstructions from handheld and robotic scans, which were completed using an existing reconstruction algorithm described in our previous work, along with the CT-to-US registration methodology^41,43,52,73^. The data are available as bone surface labels in the US seg folder, a reconstruction point cloud with outliers in ply format, and a reconstruction point cloud after removing outliers in (.stl) format.

### Robotic platform

With low-level force control, the contact force between the US probe and the patient’s back is regulated to a constant value, i.e., 5 N. Due to human breathing or involuntary patient movement, the interaction force might change, so the low-level controller would change the probe position to follow the desired contact force. The developed US scanning software includes maximum scanning velocity (4 mm/s) and maximum contact force (20 N). The robotic scanning took approximately 3.56 minutes. The maximum time was around 5 minutes, during which the scan covered three to four lumbar vertebrae on the patient’s back.

## Usage Notes

Our objective was to provide the research community with a readily accessible repository comprising a comprehensive dataset of HUS, RUS, unsegmented, and segmented CT data, along with corresponding demographics and clinical metadata from volunteers^54^. For users interested in downloading the entire dataset, a bulk download can be facilitated using the Python script available at https://www.kuleuven.be/rdm/en/rdr/large-downloads. CT data can be accessed in the publicly available open-source platforms 3D Slicer (https://www.slicer.org) or meshlab (https://www.meshlab.net/) for processing and editing 3D triangular meshes. We can also recommend the Imfusion Suite Software for image analysis of the CT and US data. For details on how data processing can be conducted, please refer to the code availability section below.

The repository currently contains 6091 expert-annotated US images from nine participants, serving as a substantial set of training images for machine and deep learning applications. The selected scans were also processed with the U-Net used in van Assche *et al*., and the resulting US reconstructions are distributed in the US_recon folder, giving an immediate baseline for future comparison^73^. Additionally, this dataset addresses several open research challenges^54^. First, robust large-scale registration of three-dimensional CT segmentations to 3D ultrasound reconstructions remains unsolved for spine applications. Second, our dataset serves as a benchmark to evaluate the generalizability of segmentation models trained on datasets like UltraBones100k to previously unseen anatomical variations^54,80^. Third, breathing-compensated *in vivo* fusion imaging is still hindered by anatomical motion. The CT-paired US sweeps released here offer ground truth for developing motion-aware correction schemes^81^. Fourth, the dataset can enable the training and testing of existing or the development of new handheld or robotic simulations based on US imaging data, with applications in a range of clinical and surgical contexts, such as the lumbar pedicle-screw placement, epidural injections, or perform anatomical measurements and spine assessments by combining precise CT surface models with co-registered ultrasound volumes^54,74,82–84^. Although the repository contains raw ultrasound data without post-processing (e.g., breathing compensation), this very limitation makes it ideally suited for algorithm development and validation in these domains^81,85,86^. Moreover, this *in vivo* dataset may serve as a valuable general resource for translational research in orthopedic machine and deep learning applications, with US shape completion efforts as previously demonstrated^54,87^.

## Data Availability

The dataset^54^ generated and described in this Data Descriptor is available at 10.48804/3XPCAE and is released under the Creative Commons Attribution 4.0 International License (CC BY 4.0; https://creativecommons.org/licenses/by/4.0/).

## Code availability

The U-Net utilized in this system was derived from the following repository: https://github.com/zhixuhao/unet. Moreover, the robotic control and data acquisition were conducted with Ubuntu (v20.04, Canonical, London, U.K.), ROS 2 (Foxy Fitzroy, Open Robotics, Mountain View, U.S.A.)^88^, and Orocos (v2.9.0, The Orocos Project, https://orocos.org/)^89^. The image processing was conducted using Python (v3.8, Python Software Foundation, Wilmington, U.S.A.), TensorFlow (v2.0, TensorFlow Developers)^90^, VTK (Kitware Inc., Clifton Park, U.S.A.), Qt 5 (Qt Group, Helsinki, Finland), and OpenCV (v3.4.2, Palo Alto, U.S.A.). It should be noted that the code of the hardware applications is considerable in length and, therefore, not feasible for sharing. However, queries can be addressed, and the code can be shared upon reasonable request.
